# Supplementary material for: Assessment of Red Blood Cell Aggregation in Preeclampsia by Microfluidic Image Flow Analysis—Impact of Oxidative Stress on Disease Severity
Source: Int J Mol Sci. 2024 Mar 27;25(7):3732. doi: 10.3390/ijms25073732 (PMC11011533; doi:10.3390/ijms25073732)
Supplement: Supplementary file 1 [file ijms-25-03732-s001.zip › ijms-2893009-supplementary.pdf]

Assessment of Red Blood Cell Aggregation in Preeclampsia by Microfluidic Image Flow Analysis—Impact of Oxidative Stress on Disease Severity

Anika Alexandrova-Watanabe [1,†], Emilia Abadjieva [1,†], Ina Giosheva [2,3], Ariana Langari [3], Tihomir Tiankov [1], Emil Gartchev [2], Regina Komsa-Penkova [4] and Svetla Todinova [3,*]

[1] Institute of Mechanics, Bulgarian Academy of Sciences, 1113 Sofia, Bulgaria
[2] University Obstetrics and Gynecology Hospital "Maichin Dom", 1431 Sofia, Bulgaria
[3] Institute of Biophysics and Biomedical Engineering, Bulgarian Academy of Sciences, 1113 Sofia, Bulgaria
[4] Department of Biochemistry, Medical University-Pleven, 5800 Pleven, Bulgaria
* Correspondence: todinova@abv.bg
† These authors contributed equally to this work.

Supplementary Data

1. Tables

Table S1. Clinical data (gestational age, GA at the time of diagnosis of PE and at delivery; mean blood pressure, BP; newborn weight, Proteinuria, PU) and biochemical indices (CRP; Platelet Count; Creatinine serum; aspartate aminotransferase, ASAT; and alanine aminotransferase, ALAT) of patients with preeclampsia (PE).

Patients' groups	Mean BP (systolic/ diastolic)	GA at diagnostic of PE	GA at delivery	Newborn weight (g)	Proteinuria (mg in 24-h urine collection)	Fg (g/L)	CRP (mg/L) (interval)	Creatinine serum (µmol/L)	ASAT (U/L)	ALAT (U/L)
PE non-severe										
Patient 1	145/93	38	38	3300	-	4.71	3.9	62	21	12.7
Patient 4	146/94	29	30	3230	647	5.85	26.87	85	66	65.9
Patient 5	145/96	33	34	2130	650	5.2	2.0	73	23	17.2
Patient 7	145/90	38	39	2860	635	5.91	3.96	80	29.7	27.3
Patient 8	148/98	38	38	3140	-	5.86	12.4	73	29	24.8
Patient11	140/90	34	34	2770	-	5.6	9.41	72	12.7	16.6
Patient12	154/105	34	34	2340	-	5.84	6.1	76	23	18.4
PE severe										
Patient 2	163/111	29	30	1389	1635	5.7	7.88	76	48.8	24.7
Patient 3	165/113	29	29	1740	1504	5.13	2.86	56	17.5	10.1
Patient 6	170/113	29	29	1200	1710	7.63	76.82	91	19	17
Patient 9	160/110	28	29	820	1785	6.28	18.56	64	11.6	11.2
Patient 10	170/114	31	31	1780	-	5.65	32.54	74	39.4	23.2
Patient 13	164/112	27	28	1400	3243	4	2.37	95	64.4	32.1

Table S2. Hematological indices (RBC count; Hemoglobin, Hb; Hematocrit, Ht; mean corpuscular volume in femtoliters (fl), MCV; mean corpuscular hemoglobin, MCH; mean corpuscular hemoglobin concentration, MCHC; red cell distribution width, RDW) and Platelet Count of patients with preeclampsia (PE).

Patients' groups	RBC count (T/L)	Hb (g/L)	Ht (L/L)	MCV (fl)	MCH (Pg/L)	MCHC (g/L)	RDW %	Platelet Count $\times 10^9$/L
PE non-severe								
Patient 1	3.96	130	0.384	92	32.8	339	12.8	277
Patient 4	3.31	122	0.319	98.1	31.3	328	10.6	243
Patient 5	3.68	110.0	0.33	90.8	30.0	346	13.7	362
Patient 7	4.26	120	0.365	87.7	29.6	339	14.6	203
Patient 8	4.23	123	0.374	93.3	30.7	329	13.6	170
Patient 11	3.79	120	0.353	96.6	33.1	340	14.4	305
Patient 12	4.33	130	0.384	88.7	30	399	15.4	324
PE severe								
Patient 2	3.86	118	0.355	92.2	30.6	330	13.3	231
Patient 3	3.85	111	0.329	85.5	28.3	332	16.6	199
Patient 6	3.8	120	0.362	95.3	31.6	331	15	212
Patient 9	4.66	128	0.393	84.4	27.5	326	11	404
Patient 10	3.5	117	0.351	100.3	33.4	333	14.2	169
Patient 13	3.53	115	0.345	97.6	32.6	333	11.4	110

Table S3. Pearson's correlation coefficient, r, calculated for the parameter pairs systolic BP/AI_L (systolic blood pressure and aggregation indices at low shear rate) and diastolic BP/AI_L (diastolic blood pressure and aggregation indices at low shear rate)

Patients' groups	r	
	systolic BP/ AI_L	diastolic BP/ AI_L
PE non-severe	0.61	0.87
PE severe	0.81	0.65

2. Figures

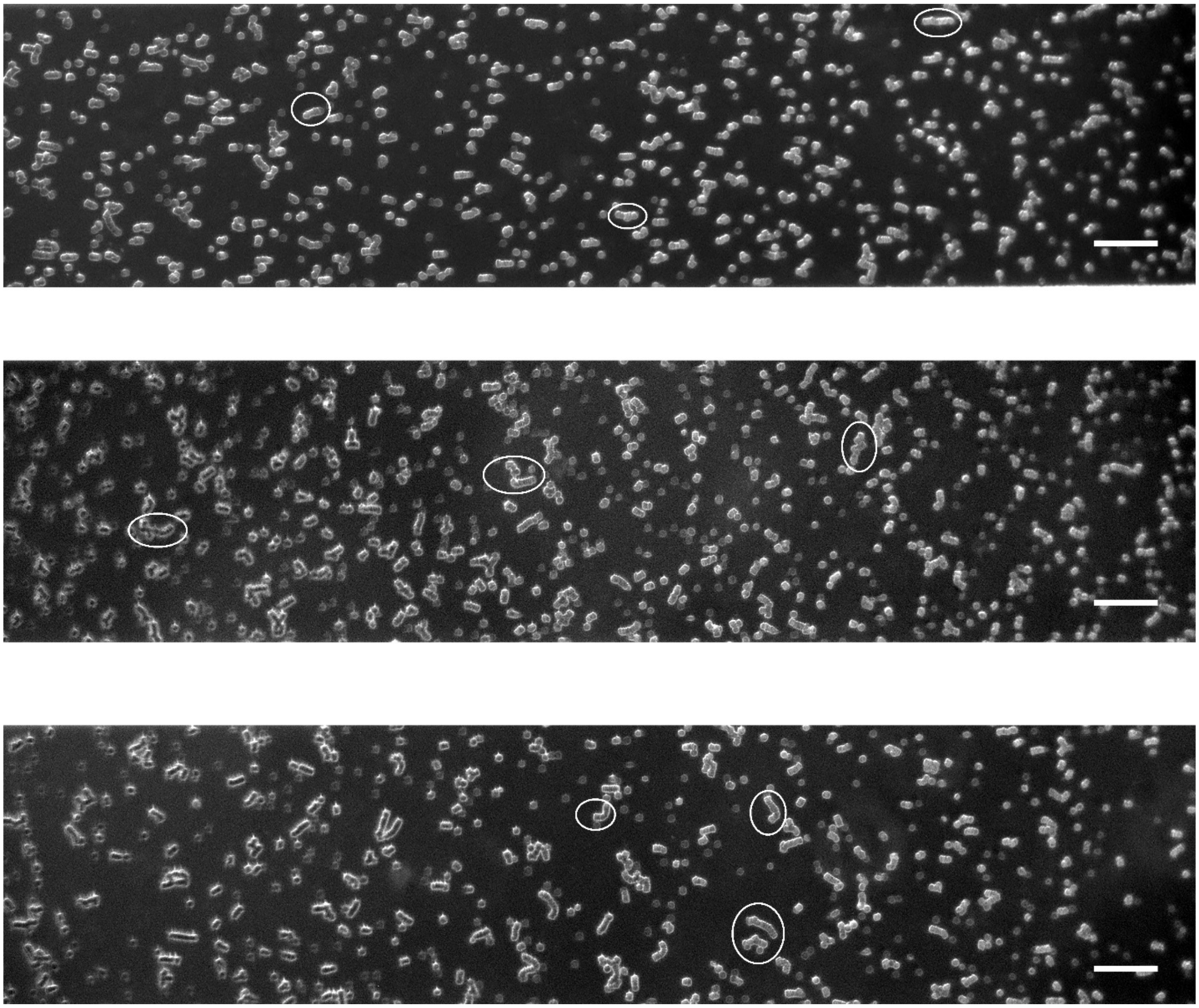

Figure S1. Groups of representative images of RBC aggregates obtained with the BioFlux microfluidic system under low-flow conditions (8.9 s^{-1}), from healthy controls (n=8). The selected images of RBCs from different healthy normotensive women show that the predominant part of the RBC aggregates represent so-called "rouleaux" formations (examples of such aggregates are encircled with white ellipses). Scale bar – 50 μm.

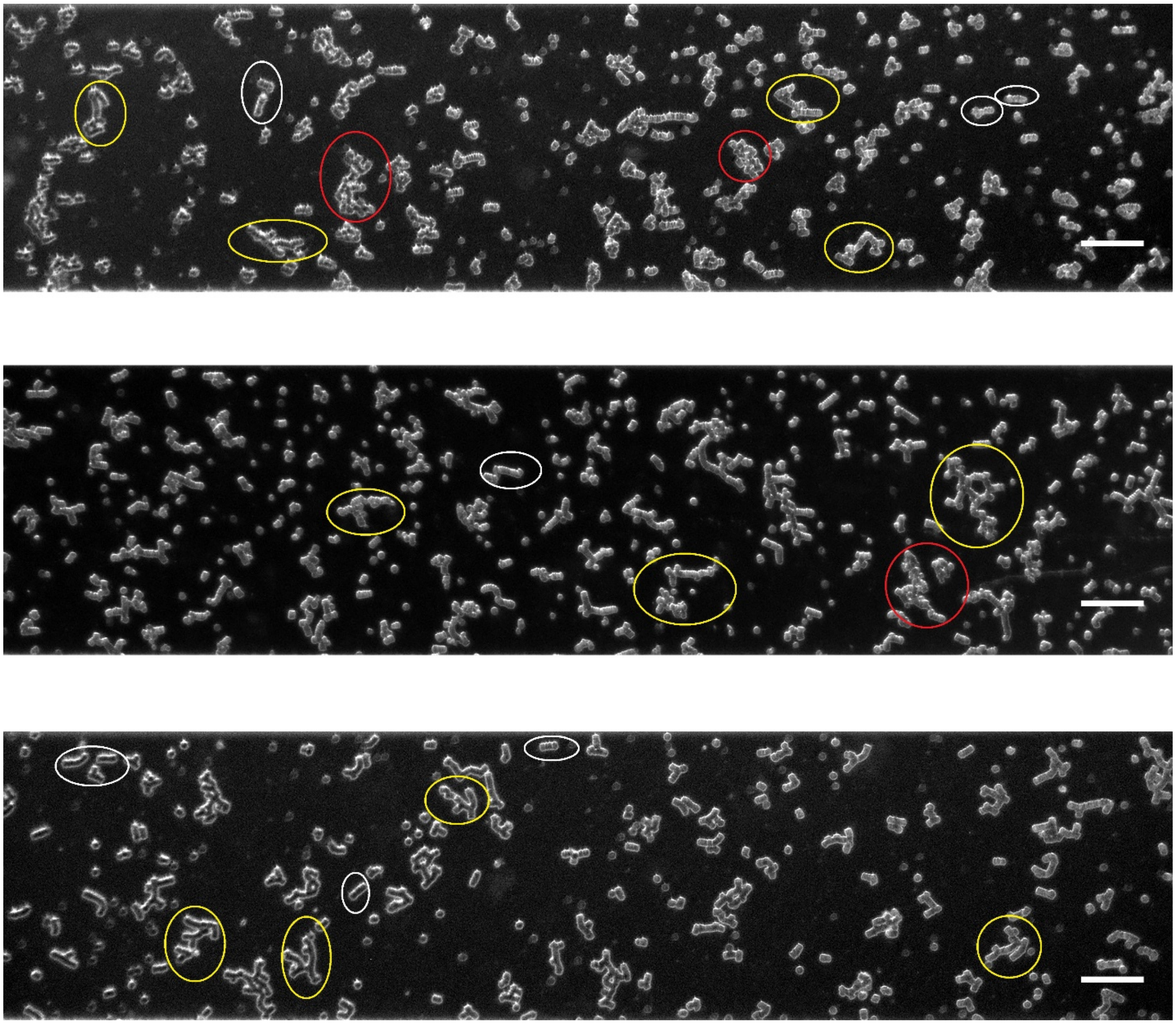

Figure S2. Groups of representative images of RBC aggregates obtained with the BioFlux microfluidic system under low-flow conditions (8.9 s^{-1}), from patients with non-severe preeclampsia (n=7). The selected images of RBCs from different patients suffering from non-severe PE demonstrate that most RBC aggregates are branched (encircled by yellow ellipses). The formation of a small amount of “rouleaux” aggregates (encircled with white ellipses), and aggregate complexes, forming networks (encircled by red ellipses) is also observed. Scale bar – 50 μm.

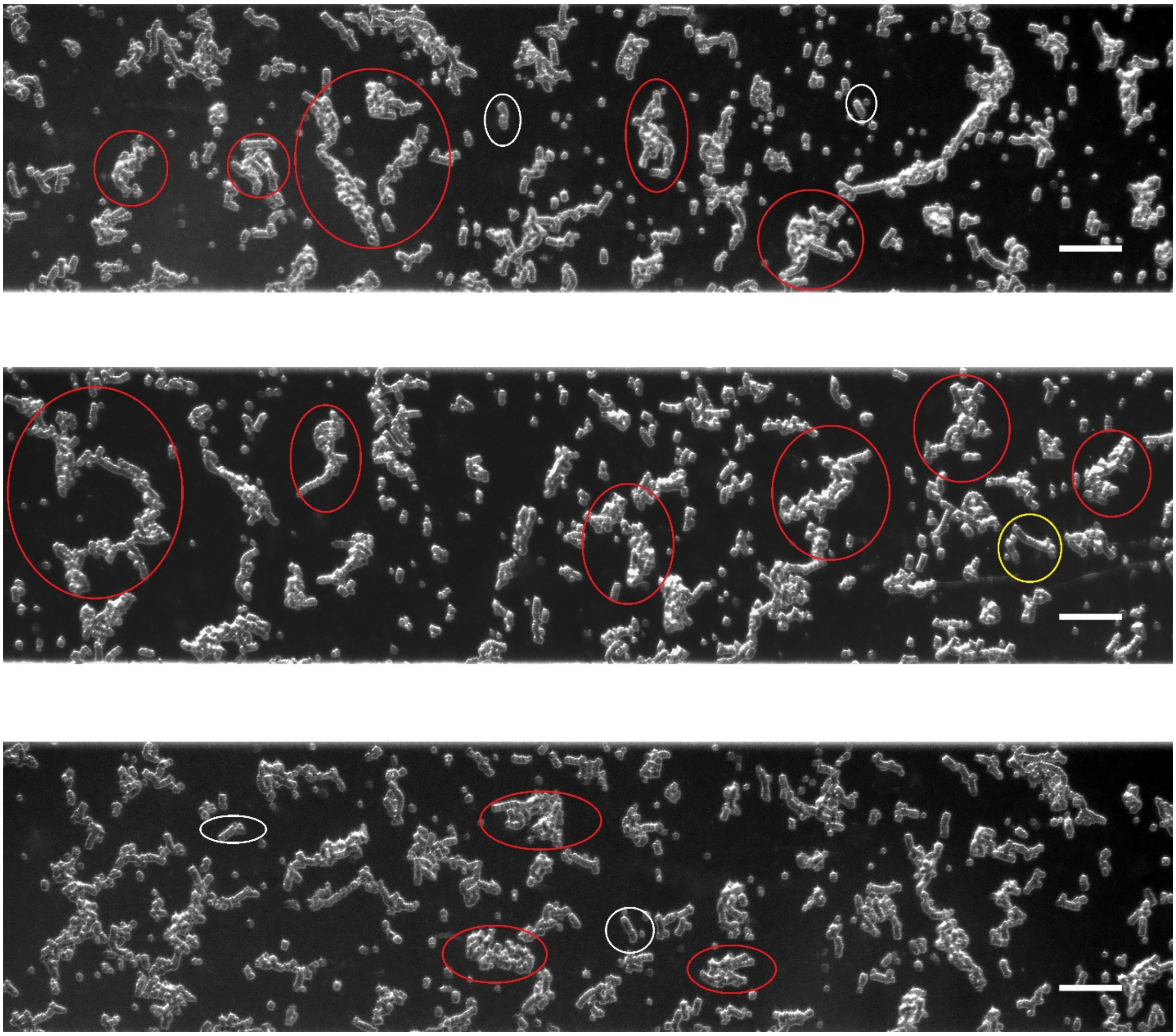

Figure S3. Groups of representative images of RBC aggregates obtained with the BioFlux microfluidic system under low-flow conditions (8.9 s^{-1}), from patients with severe preeclampsia (n=6). The selected images of RBCs obtained from different patients with severe PE demonstrate that the large aggregate complexes forming networks are prevailed (examples of such type of aggregates are encircled with red ellipses). A small part of the aggregates are "rouleaux" formations (encircled by white ellipses) and branched aggregates (encircled by yellow ellipses). Scale bar – 50 μm.

3. Methods

Quantitative Evaluation of number RBC aggregates (NA)

Computational image analysis for the evaluation of the RBC aggregates is realized as follows:

- The first step of the computational analysis is to import the obtained raw images of RBC aggregates as input data into the Image J software environment.

- Second, the *brightness/contrast tool* of the software is applied to improve the contrast of the images.

- The next step includes the application of thresholding for segmentation: it involves software automatic setting of the image's brightness levels to segregate the RBC aggregates from the rest of the image.

- The next step is the selection of the criteria parameter (pixels corresponding to aggregates including 3 cells, combined/stuck one over the other), which determines the separation between erythrocyte aggregates and non-aggregates. Based on this parameter, the initial assessment of RBC aggregates is performed. It verifies that all the RBC aggregates are selected.

- If this parameter is fulfilled/met, the next step is to obtain the table with results for the size of the area of each object of interest and their number (i.e., aggregates).

- The obtained results for the number and the area size of the RBC aggregations (total numbers and size of RBC aggregates) were calculated in a program elaborated in IntelliJ IDEA.
